# A five-year (2017–2021) time series evaluation of patient-reported informal healthcare payments in Romania

**DOI:** 10.25122/jml-2023-0080

**Published:** 2023-03

**Authors:** Ilaria Mosca, Constantin Radu, Ștefan Strilciuc, Marius-Ionuț Ungureanu

**Affiliations:** 1 Tagliente, Odijk, The Netherlands; 2 RoNeuro Institute for Neurological Research and Diagnostic, Cluj-Napoca, Romania; 3 Department of Neuroscience, Iuliu Hațieganu University of Medicine and Pharmacy, Cluj-Napoca, Romania; 4 Department of Public Health, Faculty of Political, Administrative, and Communication Sciences, Babeș-Bolyai University, Cluj-Napoca, Romania; 5 Center for Health Workforce Research and Policy, Faculty of Political, Administrative, and Communication Sciences, Babeș-Bolyai University, Cluj-Napoca, Romania

**Keywords:** Romania, patient-reported informal payments, universal health coverage, out-of-pocket payments, Patient Feedback System

## Abstract

Low wages of health professionals are widely recognized as one of the drivers of informal payments in Romania’s healthcare system. In January 2018, the government increased wages by an average of 70% to 172% in the public healthcare sector. This study examined the trends in patient-reported informal healthcare payments, discussing the effect of a one-time wage increase in 2018 and the impact of the COVID-19 pandemic in 2020 and 2021. It draws on monthly survey data of patient-reported informal payments collected between January 2017 and December 2021. We analyzed three periods: before the wage rise (“low pay”), between the wage rise and the COVID-19 pandemic (“high pay”), and during the COVID-19 pandemic. We found that patient-reported informal payments decreased between the “low pay” and “high pay” period but with a sharper decline during the COVID-19 pandemic. The share of respondents willing to report informal payments increased during the “high pay” period, indicating a stronger willingness to voice dissatisfaction with health services and informal payments, but slowed down during the first lockdown in 2020. Informal payments were more frequently reported in larger hospitals and the poorest geographical areas. While the 2018 wage increase may have contributed to less prevalent informal payments, survey coverage and design must be improved to draw robust, system-level conclusions to inform tailored policy actions.

## INTRODUCTION

Universal health coverage (UHC) is one of the 17 Sustainable Development Goals, aimed at ensuring that everyone has access to quality healthcare services without experiencing financial hardship. People can experience financial hardship when they are required to make out-of-pocket payments that are significant in proportion to their ability to pay for healthcare. Out-of-pocket payments can be formal – these include user charges or fees for covered goods and services as well as payments for the private purchase of goods and services, or informal. Informal payments are “a direct contribution made in addition to any contribution determined by the terms of entitlement, in cash or in kind, by patients or others acting on their behalf, to healthcare providers for services to which patients are entitled” [1]. These payments can take many forms, including in-kind payments in the form of (gratitude) gifts and cash payments outside the official payment channels.

Informal payments are widely prevalent in many countries and have significant negative impacts on health systems [2,3]. This issue has been extensively studied in various developing economies, including Central and Eastern European countries [4-7]. Informal payments skew the resources distribution and set barriers to equitable access to affordable and high-quality health services. As a result, people who pay informally are pushed into (or even below) poverty, reducing their demand for care and foregoing access to health services [8-11]. Poor people, regular users of health services, and people with chronic conditions are among the most affected by informal payments [2, 12].

Several factors - in and outside the health system - are responsible for informal payments. Factors outside the health system include the overall level of corruption in a country, the weak governance of a country’s institutions, and a general social and cultural acceptance of informal payments. Health-system factors are linked with the low overall public spending on health, decentralization of funds pooling, unclear entitlements to health benefits, outdated provider payment systems that do not pay for performance, low quality of health services and goods, and limited access to publicly funded health services [13].

There is a wide variety of strategies to deal with informal payments, which can cover three areas: cultural perceptions, insufficient funding of the health sector, and lack of control and accountability in the health system [2,13]. It is important to note that there is no "one size fits all" approach to addressing informal payments, and strategies must be tailored to the specific context. For example, a study that examined informal payment patterns across countries found that introducing a formal co-payment system successfully lowered informal payments in Bulgaria but not in Hungary, where formal user charges were largely opposed and quickly abolished soon after their introduction [6].

Our article focused on informal payments for hospital services in Romania. Atenţie is the Romanian word used to define informal payments in the form of bribes, gifts, or services. Atenţii have long been a part of the Romanian health system, and they kept it functional in the context of underfunding and low wages of healthcare workers in the public sector [4]. In 2004, informal payments were estimated at around €300 million [14]. The ASSPRO CEE 2007 report estimated that almost half of adult people pay informally for inpatient care, and about 29% do so for outpatient care. The average informal payment for inpatient and outpatient care was €63 and €15, respectively. Informal payments as a share of total health spending were estimated at 6.3%, one of the highest shares among Central and Eastern European countries [15]. According to a Special Eurobarometer report on corruption, Romania has one of the highest reported rates of informal payments in the EU, with 20% of survey respondents who visited a public healthcare provider in the previous 12 months reporting having to make an extra payment, give a valuable gift to a nurse or doctor, or make a donation to the hospital. This is far above the EU average of 4%, placing Romania at the top of the EU countries [16]. In 2015, 12.5% of households in Romania experienced catastrophic levels of formal and informal health spending, up from 10.4% in 2010 [17].

The low public spending on health in Romania has led to the widespread dual practice of physicians working in both the public and private health sectors, allowing doctors to open their private practices for higher income. Palaga describes the dual practice as “triggering power relations that lead to exclusion and layering,” with patients from public institutions redirected to private clinics [18]. Through dual practice, informal payments are replaced by referrals to the private sector, where the treating physician is either a practitioner or shareholder. People pay extra in private practices for health services they are entitled to under the public system [18]. In essence, informal payments are “formalized” via the physicians’ dual practice.

In March 2018, the Romanian Government implemented a significant increase in wages for public healthcare professionals, in an attempt to bring their income levels closer to the EU average while curbing systemic challenges such as brain drain or informal payments. The measure impacted most staff categories, except for biologists, chemists, biochemists, and auxiliary staff, with resident physicians benefitting from up to a fourfold salary increase.

The goal of this study is to understand the impact of the one-time wage increase in 2018 and the COVID-19 pandemic in 2020 and 2021 on patient-reported informal payments in publicly funded hospitals in Romania.

## MATERIAL AND METHODS

The National Patient Feedback System (NPFS) has been operational since December 2016 and includes 351 public hospitals in Romania, covering the entire public hospital network except for the 21 military and penitentiary hospitals. The NPFS has ten questions with an overall focus on patient satisfaction at hospital discharge. The last two questions inquire about informal payments, in particular, “Have nurses or doctors asked you for bribes?” (Q9) and “Do you want to report being asked for bribes to the anti-corruption officer at the Ministry of Health?” (Q10). Both questions are closed, i.e., “Yes” or “No” questions. The questionnaire is delivered to patients via text message, with patients having previously expressed their consent to be contacted by the hospital where they received care. Patients can respond to the survey by either replying to the text message or online.

We performed a time series evaluation of patient-reported informal healthcare payments in Romania by analyzing publicly available data from the NPFS. The data were downloaded from the Romanian Ministry of Health website in JSON format and imported into Microsoft Excel. The resulting dataset spans five years (Jan 2017 to Dec 2021). During the first two years since it was implemented, the NPFS encountered significant technical issues in October 2017 and June 2018. Due to the low number of patient feedback questionnaires collected, these two months were excluded from our analysis.

Public hospitals in Romania commonly send out electronic feedback questionnaires to varying proportions of their patients. To limit parameter uncertainty when drawing conclusions, we used two filters to remove insufficient or otherwise biased aggregate entries. The first filter ensured significance for every statistical unit and consisted of a numeric minimum threshold of 30 monthly responses per hospital. The second filter aimed to increase the representativity of results by removing hospitals that lacked consistency in sending out the feedback questionnaires and consists of the annual share of answers to Q9 out of the number of inpatient cases for each hospital averaged for the entire 5-year period (Q9-IC). Due to the right-skewed distribution of the Q9-IC indicator (min=0, max=44.1, mean=5.5, median=3.2, standard deviation=6.7, skewness=2.2, kurtosis=6.2), its threshold was set to its first quartile value (1.1%). Hospitals with a Q9-IC value below this threshold were excluded from the analysis (n=85). Data on the number of inpatient cases for all public hospitals were downloaded from the National School of Public Health website. The two datasets (patient feedback and yearly volume of inpatient cases) were matched using unique IDs for each hospital. The Q9-IC indicator could not be calculated for hospitals included in the NPFS with no data on inpatient cases (n=12), leading to their removal from our analysis. The final number of hospitals analyzed was 254.

The analysis was divided into three distinct periods: before the salary increase (“low pay” – Jan 2017 to Dec 2017), after the salary increase and up until the start of the COVID-19 pandemic (“high pay” – Jan 2018 to Feb 2020) and after the debut of the pandemic (“COVID” – Mar 2020 to Dec 2021). Hospitals were also classified into three size categories (small, medium, and large) based on the distribution of the yearly average number of inpatient cases for the 2017–2021 period. Given that the distribution was highly right-skewed (min=95, max=49705, mean=8880, median=4784, standard deviation=10068, skewness=1.9, kurtosis=3.2), the upper bound of the “small” category was set to the median value (rounded to the nearest thousand), and the upper bound of the “medium” category was set to the third quartile value (rounded to the nearest thousand). Hospitals with a yearly average number of inpatient cases above the upper bound of the “medium” category were included in the “large” category.

The COVID-19 status of hospitals was determined based on their overall involvement in handling COVID-19 patients throughout the pandemic. As of April 2020, the Order of the Minister of Health no. 555/2020 and its subsequent modifications defined types of COVID hospitals based on their degree of involvement. Phase I hospitals were primarily infectious disease hospitals and handled COVID-19 cases exclusively, while Phase II hospitals had a share of their wards reserved for COVID-19 patients. For this reason, hospitals were categorized as COVID-19 based on their Phase I and Phase II status at the beginning of the pandemic.

Hospitals were split according to their governing authority: clinical institutes under the authority of the Ministry of Health, county hospitals under the authority of county councils, and municipal hospitals under the authority of city halls and local councils. Hospitals not included in the previously mentioned categories were categorized as “Other”. In general, the latter category contains either small-town hospitals or hospitals operating under separate networks (i.e., Ministry of Transport).

Romania is divided into 41 counties, including the capital city Bucharest, and 8 development regions, encompassing between four and seven counties. As these divisions have broad implications in resource allocation, hospitals in our analysis were additionally grouped by county and development region. The final dataset consisted of the monthly “Yes” and “No” answers to Q9 and Q10 for each hospital adjusted for all hospital characteristics. The dataset was analyzed in Microsoft Excel and Tableau Desktop.

## RESULTS

Our analysis covers 254 public hospitals in Romania, about 72% of the public hospitals included in the NPFS. Table 1 presents the characteristics of the hospitals.

**Table 1:** Characteristics of hospitals.

Characteristics and categories	Number of hospitals
**Size**	
Small	116
Medium	61
Large	77
**Type**	
County	37
Municipal	49
Institute	18
Other	150
**COVID-19 status**	
COVID-19	30
Non-COVID-19	224

The number of patients who answered the feedback questionnaire sent by the hospitals registered an overall increase over the 5-year time span, with a decrease recorded only in 2020 at the start of the COVID-19 pandemic. The sample size for Q9 grew from 94,025 in 2017 to 197,219 in 2021 (+210%). For Q10, the sample size was 91,295 in 2017 and increased to 193,369 in 2021 (+212%).

**Figure 1:**
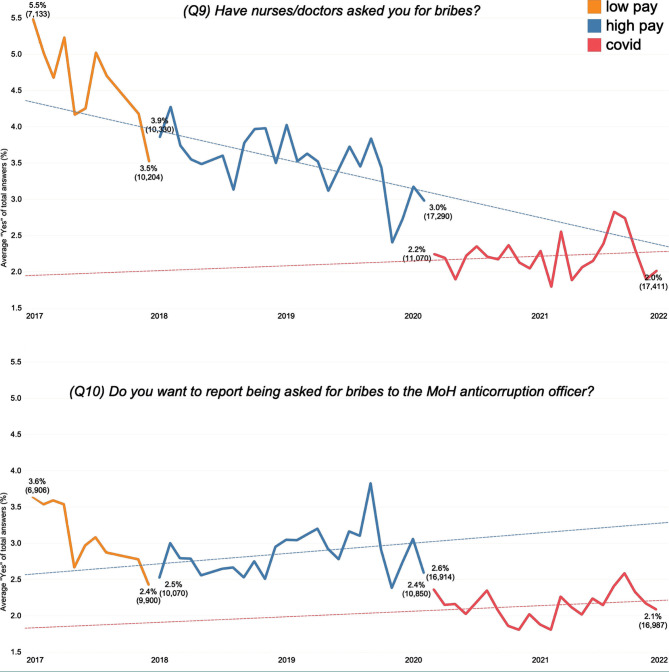
Monthly average “Yes” answers to Q9 and Q10, by period. Brackets show the number of answers corresponding to the respective percentage. MoH – Ministry of Health.

The average percentage of “Yes” answers for the entire 5-year period was 3.2% for Q9 (95% confidence interval: 3.0%-3.5%) and 2.6% for Q10 (95% confidence interval: 2.5%-2.8%). The monthly average percentage of “Yes” answers to Q9 decreased steadily between 2017 and the first months of 2020, with a relatively smooth transition between the “low pay” and “high pay” periods. A much sharper decrease occurred at the beginning of the COVID-19 pandemic in March 2020, and then the percentage fluctuated and peaked towards the end of 2021. As for Q10, there is an almost seamless transition of the percentage of “Yes” answers between the “low pay” and “high pay” phases. With the start of the pandemic, the monthly average percentage of “Yes” answers for Q10 gradually decreased, albeit less significantly than Q9 (Figure 1).

**Figure 2:**
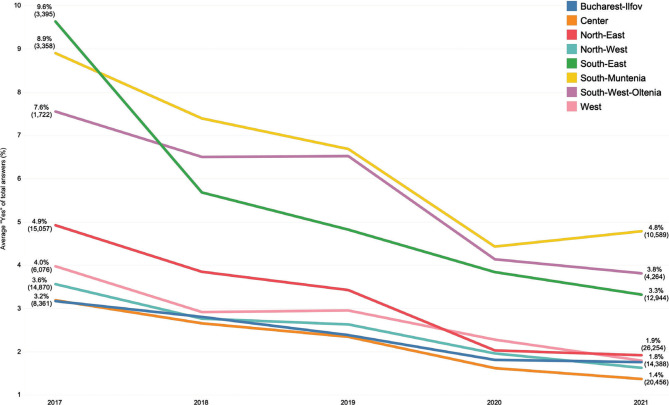
Monthly average “Yes” answers to Q9, by development region. Brackets show the number of answers corresponding to the respective percentage.

The regions South-East, South-Muntenia, and South-West had, on average, 5.9% of patients reporting having made informal payments (Q9) (95% confidence interval: 4.8%-6.9%) over the 5 years, compared to all other five regions with an average of 2.6% (95% confidence interval: 2.3%-3.0%) (Figure 2). These three regions are among the poorest in Romania, suggesting a positive correlation between poverty and informal payments. The same three southern regions had the highest monthly average of patients willing to report informal payments (Q10), although the difference with the other development regions was less marked than for Q9.

**Figure 3:**
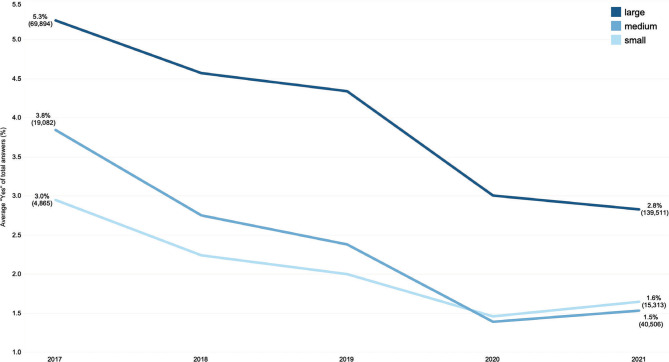
Monthly average “Yes” answers to Q9, by hospital size. Brackets show the number of answers corresponding to the respective percentage.

Larger hospitals had a higher monthly average share of people reporting making informal payments (Q9) (Figure 3). The average share over the 5-year time span was 4% (95% confidence interval: 2.7%-5.3%) for large hospitals, while medium-sized and smaller hospitals had an average of 2.2% (95% confidence interval: 1.7%-2.8%). It is also worth noticing that once the pandemic began, smaller hospitals replaced medium-sized hospitals when ranked by the monthly average percentage of “Yes” answers to Q9. A very similar behavior was observed for Q10, though the gap between the three types of hospitals is much smaller.

The number of answers to both questions increased from one year to another for all hospital types, except in 2020 (Table 2). In 2017 and 2018, more patients answered Q9 than Q10, but this trend reversed at the beginning of 2019, especially for institutes compared to other hospital types.

**Table 2:** Sample sizes and percentages of “Yes” answers for Q9 and Q10, by year and hospital type.

Year	Question	Indicator	Hospital type			
			County	Municipal	Institute	Other
2017	Q9	Avg. %Yes of total	6.7%	7.1%	2.4%	3.1%
		Sample size	20.740	9.417	21.515	42.169
	Q10	Avg. %Yes of total	4.0%	4.2%	2.1%	2.4%
		Sample size	20.209	9.090	20.981	40.838
	Percentage difference between no. of answers to Q10 and Q9		-0.42%	-0.42%	-0.18%	-0.25%
2018	Q9	Avg. %Yes of total	6.1%	5.0%	2.0%	2.5%
		Sample size	35.029	14.141	37.033	66.766
	Q10	Avg. %Yes of total	4.0%	3.4%	1.9%	2.0%
		Sample size	34.170	13.596	36.179	65.185
	Percentage difference between no. of answers to Q10 and Q9		-0.36%	-0.34%	-0.09%	-0.22%
2019	Q9	Avg. %Yes of total	5.8%	4.4%	1.9%	2.1%
		Sample size	45.232	19.023	44.460	85.180
	Q10	Avg. %Yes of total	4.4%	3.8%	2.1%	2.2%
		Sample size	44.152	18.525	43.489	83.261
	Percentage difference between no. of answers to Q10 and Q9		-0.26%	-0.15%	0.08%	0.03%
2020	Q9	Avg. %Yes of total	3.9%	2.8%	1.4%	1.5%
		Sample size	34.345	13.534	30.395	62.978
	Q10	Avg. %Yes of total	3.1%	2.6%	1.6%	1.7%
		Sample size	33.666	13.168	29.828	61.759
	Percentage difference between no. of answers to Q10 and Q9		-0.21%	-0.12%	0.12%	0.12%
2021	Q9	Avg. %Yes of total	3.8%	2.6%	1.0%	1.6%
		Sample size	52.052	20.556	38.970	83.752
	Q10	Avg. %Yes of total	2.9%	2.7%	1.5%	1.8%
		Sample size	51.056	20.140	38.167	82.156
	Percentage difference between no. of answers to Q10 and Q9		-0.23%	0.03%	0.45%	0.07%

When analyzing the hospital types, county hospitals had the highest monthly average percentage of “Yes” answers to Q9, followed by municipal hospitals, while institutes had the lowest monthly average percentage of people reporting paying informally. The timeline for Q10 follows an almost identical pattern (Table 2).

**Figure 4:**
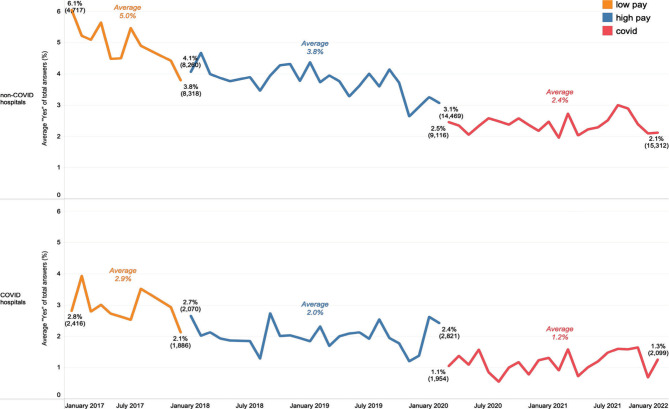
Monthly average “Yes” answers to Q9, by hospital COVID status and period. Brackets show the number of answers corresponding to the respective percentage.

COVID-19 hospitals registered a lower average percentage of “Yes'' answers to Q9 during the pandemic than non-COVID hospitals (Figure 4). However, this was also the case before the pandemic, even though the drop in the percentage of “Yes'' answers was more abrupt for COVID-19 hospitals, likely due to the disruption of elective surgeries and other planned activities. The above-described pattern is almost identical for Q10, except that the transition between the pre-pandemic and pandemic phases is much smoother for both COVID-19 and non-COVID-19 hospitals.

## DISCUSSION

The introduction of the survey with questions on informal payments for health services is an important first step in monitoring the evolution of this issue over time. While collecting data through the survey is essential in understanding the trend of informal payments in the healthcare system, it is equally important to systematically analyze and monitor the data to provide insights for policymaking. The potential of this instrument could be further enhanced with some changes in the design of the survey.

The current dataset does not allow measuring and monitoring the frequency and the magnitude of informal payments, nor does it allow us to understand for which inpatient services people spend the most out of their pocket, i.e., if it is informal payments to health professionals or to receive better quality care/amenities or both. It would also be beneficial to collect additional information on the socioeconomic characteristics of the respondents.

The survey is likely to be subject to selection/sampling bias that skews the findings because feedback is captured from a certain segment of the audience. People might be reluctant to provide answers due to the sensitive nature of the topic, fear of repercussions, the method of delivery (text message/online), and (lack of) trust in the system’s capacity to change anything based on feedback forms and data. An additional source of bias in our results may be related to respondents’ unwillingness to report a negative hospital interaction or the other way around.

While our study shows a reduction in the prevalence of informal payments for healthcare services following the 2018 wage increase, it is challenging to isolate the impact of the wage increase alone. However, an “unconditional” pay rise is unlikely to be an effective long-term strategy to tackle informal payments, as its effect wears off over time. Better ways to increase health workers’ income should be linked to increased performance, both in terms of efficiency and responsiveness to patients’ expectations [2]. To ensure equitable access to healthcare, which can be hindered by informal payments, complementary policies should be implemented, such as revising the positive list of drugs, redesigning the basic benefits package, increasing public funds and the government's prioritization of health, and stronger enforcement of sanctions for doctors who accept informal payments.

Effectively addressing informal payments in healthcare requires a tailored approach that takes into account the unique context of the country. In Romania, informal payments, known as atenţii, are deeply ingrained in the culture. The fact that an increasing number of people have reported making informal payments in recent years highlights a growing dissatisfaction with the current state of the healthcare system and the services it provides.

## CONCLUSION

Although informal payments in Romania have decreased in recent years due to a one-time wage increase in the public sector and the COVID-19 pandemic, they still remain a significant obstacle to accessing healthcare services. Systematic data monitoring, changes to the survey design, and complementary policies to better manage informal payments could contribute to improved access to health services and better financial protection.

## References

[1] Cherecheş RM, Ungureanu MI, Sandu P, Rus IA (2013). Defining informal payments in healthcare: a systematic review. Health Policy.

[2] World Health Organization ROfE (2010). Implementing health financing reform: lessons from countries in transition.

[3] Pavlova M Assessment of patient payment policies and projection of their efficiency, equity and quality effects: The case of Central and Eastern Europe.

[4] Buligescu B, Peňa HE (2020). Informal payments in Romanian health care system. A sample selection correction 1.

[5] Gercheva S The controversial health care reform in Bulgaria: Financial sustainability of health insurance twenty years on. Economic Science, Education and the Real Economy: Development and Interactions in the Digital Age.

[6] Stepurko T, Pavlova M, Gryga I, Gaál P, Groot W (2017). Patterns of informal patient payments in Bulgaria, Hungary and Ukraine: a comparison across countries, years and type of services. Health Policy Plan.

[7] Tomini SM, Groot W, Pavlova M, Tomini F (2015). Paying Out-of-Pocket and Informally for Health Care in Albania: The Impoverishing Effect on Households. Front Public Health.

[8] Falkingham J (2004). Poverty, out-of-pocket payments and access to health care: evidence from Tajikistan. Soc Sci Med.

[9] Mæstad O, Mwisongo A (2011). Informal payments and the quality of health care: Mechanisms revealed by Tanzanian health workers. Health Policy.

[10] Stepurko T, Pavlova M, Gryga I, Groot W (2013). Informal payments for health care services–Corruption or gratitude? A study on public attitudes, perceptions and opinions in six Central and Eastern European countries. Communist Post-Communist Stud.

[11] Polese A (2014). Informal payments in Ukrainian hospitals: On the boundary between informal payments, gifts, and bribes. Anthropol Forum.

[12] Constanta M-P, Silvia F (2013). Direct patient payments in Romania: between burden and willingness to pay – a quantitative study. National School of Public Health, Management and Professional Development.

[13] Zandian H, Esfandiari A, Sakha MA, Takian A (2019). Strategies to reduce informal payments in health systems: a systematic review. East Mediterr Health J.

[14] Cherecheş RM, Ungureanu MI (2012). The saga of the Romanian health-care reform. Lancet.

[15] European C (2013). Formal and informal out-of-pocket payments for health care services in Central and Eastern European countries. What are the actual patients’ contributions? Brussels.

[16] European C (2017). Corruption - Eurobarometer survey.

[17] Scîntee G, Mosca I, Vlădescu C (2022). Can people afford to pay for health care?. New evidence on financial protection in Romania. Copenhagen: WHO Regional Office for Europe.

[18] Palaga C (2015). From informal exchanges to dual practices. The shadows of the Romanian health care reform. Stud Univ Babes-Bolyai Sociol.

